# Mapping the 3D structures of small molecule binding sites

**DOI:** 10.1186/s13321-016-0180-0

**Published:** 2016-12-06

**Authors:** Joshua Meyers, Nathan Brown, Julian Blagg

**Affiliations:** Cancer Research UK Cancer Therapeutics Unit, Division of Cancer Therapeutics, The Institute of Cancer Research, London, SM2 5NG UK

**Keywords:** Binding site prediction, Binding site comparison, Mapping binding site space, Protein structure

## Abstract

**Background:**

Analysis of the 3D structures of protein–ligand binding sites can provide valuable insights for drug discovery. Binding site comparison (BSC) studies can be employed to elucidate the function of orphan proteins or to predict the potential for polypharmacology. Many previous binding site analyses only consider binding sites surrounding an experimentally observed bound ligand.

**Results:**

To encompass potential protein–ligand binding sites that do not have ligands known to bind, we have incorporated fpocket cavity detection software and assessed the impact of this inclusion on BSC performance. Using fpocket, we generated a database of ligand-independent potential binding sites and applied the BSC tool, SiteHopper, to analyze similarity relationships between protein binding sites. We developed a method for clustering potential binding sites using a curated dataset of structures for six therapeutically relevant proteins from diverse protein classes in the protein data bank. Two clustering methods were explored; hierarchical clustering and a density-based method adept at excluding noise and outliers from a dataset. We introduce circular plots to visualize binding site structure space. From the datasets analyzed in this study, we highlight a structural relationship between binding sites of cationic trypsin and prothrombin, protein targets known to bind structurally similar small molecules, exemplifying the potential utility of objectively and holistically mapping binding site space from the structural proteome.

**Conclusions:**

We present a workflow for the objective mapping of potential protein–ligand binding sites derived from the currently available structural proteome. We show that ligand-independent binding site detection tools can be introduced without excessive penalty on BSC performance. Clustering combined with intuitive visualization tools can be applied to map relationships between the 3D structures of protein binding sites.

**Graphical abstract:**
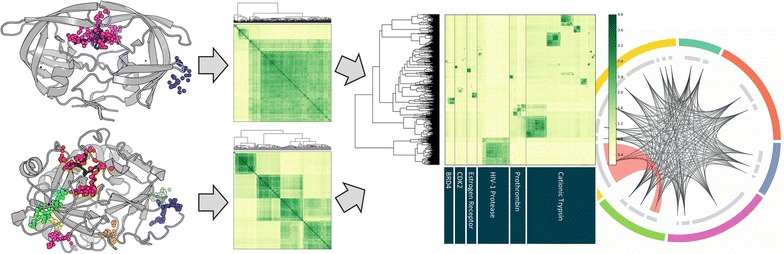
Mapping binding site space.

**Electronic supplementary material:**

The online version of this article (doi:10.1186/s13321-016-0180-0) contains supplementary material, which is available to authorized users.

## Background

Analysis of the three-dimensional structures of proteins is integral to our understanding of the molecular machinery involved in their biological function and is increasingly enabled by the wealth of structural data available in the Protein Data Bank (PDB) [1]. In particular, the examination of functional binding sites is of importance in biological chemistry and drug discovery by rational design [2]. Here, we present a method for generating a structural map of potential small molecule binding sites derived from the currently available structural proteome [3, 4].

Evidence for the existence and location of a binding site can be built through experimental observation of protein–ligand binding events—often facilitated by protein X-ray crystallography and/or Nuclear Magnetic Resonance (NMR) spectroscopy. However, prospective computational analysis to discover novel potential binding sites requires an objective and systematic cavity detection method, for which many tools exist [5–7]. For example, *fpocket* is a widely used and freely available software that employs geometric alpha shape theory to detect cavities in protein structure coordinates [8].

**Fig. 1:**
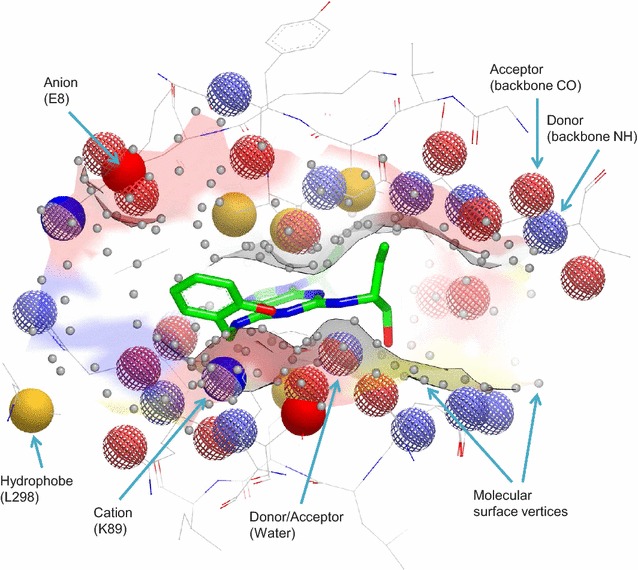
SiteHopper patch exemplified for the cofactor binding site of CDK2 (PDB ID: 2A0C) defined by surface protein atoms within 4 Å of a bound ligand (shown in *green*). A pharmacophore model defines pseudocenters for five key interaction types: hydrogen bond donor (*blue mesh*), hydrogen bond acceptor (*red mesh*), anion (*red solid*), cation (*blue solid*) and hydrophobe (*yellow*). Surface vertices also encode the shape of the binding site (*gray*). Image produced using VIDA [17]

A number of *Binding Site Comparison* (BSC) tools exist to quantify the structural similarity between a pair of binding sites [9–12]. BSC has been applied to suggest the function of orphan proteins and to predict the potential for polypharmacology [4, 12]. *SiteHopper* [13] is a recently developed BSC tool in which binding sites are represented as three-dimensional *patches* encoded with spatial information concerning the local molecular surface (shape) and chemical properties (color) of residues lining the binding site. An example binding site patch is shown in Fig. 1 for the cofactor binding site of CDK2 [14]. Binding site patches can be aligned rapidly in a pairwise manner and assessed for structural similarity based on the maximal overlap of atom-centered Gaussian functions [15]. A more detailed description of the SiteHopper BSC tool has recently been published [16].

BSC tools commonly define binding sites as the protein environment surrounding an experimentally observed bound ligand. Importantly, this definition excludes potential binding sites that have not been demonstrated to bind ligands (so called *unliganded* binding sites), thereby creating a bias towards currently exemplified protein–ligand complexes. To address this, tools such as *CavBase* [18], *RAPMAD* [19], *IsoMIF* [20] and *TrixP* [21], have integrated binding site detection algorithms with BSC. However, to the best of our knowledge, there has been no systematic analysis of the implications for BSC performance with unliganded cavities in the dataset. To mitigate this concern, we applied a modular approach and independently validated both the cavity detection and BSC components when applied to datasets comprising both liganded and unliganded protein binding sites.

A structural mapping of protein binding sites can provide a useful tool for probing the three-dimensional structural relationships between biological macromolecules [3, 10]. Tools that aim to provide an assessment of similarity between protein–ligand binding sites include *Relibase*, a database of known protein–ligand binding sites [22]; the *sc*-*PDB*, an annotated database of druggable binding sites from the PDB [23]; and the *Pocketome*, an encyclopedia of conformational ensembles of druggable binding sites [24]. While each of these tools provides an assessment of similarity between binding sites, the potential for identifying novel three-dimensional relationships involving currently unliganded binding sites is limited without the incorporation of objective methods for cavity detection.

The workflow presented here enables a structural mapping of potentially ligandable binding sites of the currently available structural proteome. We apply fpocket to objectively detect protein cavities and SiteHopper BSC to systematically generate pairwise structural similarities between all detected cavities. We also assess the performance of BSC incorporating all fpocket-detected cavities versus datasets only containing cavities surrounding an experimentally observed ligand. We then describe a number of clustering methods and visualization techniques for the mapping of potential binding site space. Altogether we present a validated workflow and describe challenges associated with the methodologies employed therein. In this work, we have adopted the following definitions throughout: a cavity is a surface depression identified by fpocket in static protein structure data. A potential protein–ligand binding site is one predicted by fpocket to bind small molecules, whereas a known protein–ligand binding site is one that has been experimentally shown to bind small molecules.

## Methods

### Protein structure datasets

Four datasets of protein structures were studied.

An ensemble of five cAMP-dependent protein kinase structures, all bound to the endogenous co-factor adenosine 5′-triphosphate (ATP) (Additional file), was retrieved from the PDB [1]. Crystallographic structure data was selected to satisfy ligand-centric quality criteria: resolution ≥2.7 ångströms (Å) [25], Real-Space Correlation Coefficient (RSCC) of ligand instance ≥0.9, Real-Space R-factor (RSR) of ligand instance ≤0.15 and Occupancy-Weighted Average B-factor (OWAB) of ligand instance between 5 and 50 Å^2^ [26]. All structures were aligned using the PDB entry 1ATP as the reference coordinates and Schrödinger’s *Protein Preparation Wizard* [27] was applied to ensure consistent protonation, removal of waters and assignment of tautomers. All structures exhibit very similar conformations of the protein with a mean all-atom Root-Mean-Square Deviation (RMSD) for pairwise alignment of 1.0 Å, calculated in PyMOL using the *align* command with the *cycles* flag set to zero [28].

The *PDBBind*-refined set (2014) [29] is a curated set of 3446 high-quality, binary protein-small molecule complexes associated with measured binding affinity (*K*_*i*_ or *K*_*d*_). This dataset was used to evaluate models for ranking detected cavities and to determine a threshold above which detected cavities constitute potential binding sites.

The *sc*-*PDB* (2013) [23] is a curated database of 9283 proteins bound to drug-like ligands and was used to assess the performance of SiteHopper BSC [13].

A further dataset was manually curated from the PDB to guide the generation of a map of the structures of diverse and therapeutically relevant potential small molecule binding sites. This dataset contains 1085 crystallographically determined protein structures of the following protein targets: bromodomain-containing protein 4 (BRD4) (93), cyclin-dependent kinase 2 (CDK2) (148), estrogen receptor (52), human immunodeficiency virus-1 (HIV-1) protease (335), prothrombin (142) and cationic trypsin (315). Structures were retrieved by their respective *UniProt* [30] identifiers, except HIV-1 Protease for which structures were retrieved with 90% sequence identity (*Protein BLAST* [31], *E* = 10^−20^) to a reference sequence [32]. Retrieved crystal structures were selected to satisfy protein-centric crystallographic quality criteria: resolution ≥2.5 Å, Free R-factor (Rfree) ≤0.3 and Diffraction Precision Index (DPI) [33] ≤0.5 Å [34]—calculated using DPI calculator [35]. This dataset is referred to as the *Pilot* dataset (Additional file).

All crystallographic quality descriptors were retrieved from either the PDB or *Electron Density Server* (EDS) [36] unless otherwise stated.

### Binding site detection

fpocket (version 2.0) [8] was implemented for ligand-independent cavity detection using default settings with two parameter alterations; the −*r* flag was set to 3.0 (default 4.5) and the −*n* flag was set to 3 (default 2). fpocket ranks cavities according to a *Partial Least Squares* (PLS) model *Score* trained on five descriptors relating to hydrophobicity, polarity and the size of a detected binding site [8]. Cavities were detected for protein structures in the PDBBind-refined set (2014) [29] and an fpocket *Score* ≥16.8 was determined, above which cavities were considered as potential ligand-binding sites. This threshold corresponds to the *Score* above which 95% of known ligand binding sites from the PDBBind-refined set were identified.

### Binding site comparison (BSC)

A dataset of ligand-dependent binding sites was generated using the SiteHopper *create* tool [13] where default parameters create a binding site patch within 4 Å of a specified bound ligand. This approach was followed to generate 9275 binding site patches for the sc-PDB (2013) database; this is referred to as the *ligCav* binding site dataset. Eight protein structures failed to yield binding site patches.

To generate ligand-independent binding site patches, surface protein atoms associated with fpocket cavities were utilized as a pseudo-ligand for input to the SiteHopper *create* tool. Binding site patches were defined as surface protein atoms lying within 0.3 Å of the fpocket surface atoms. This *site size* value was determined empirically through a number of retrieval experiments with a range of *site size* values (0.1–0.6 Å, increments of 0.1 Å). The ability of SiteHopper to identify similarity between a query estrogen receptor binding site patch and other members of the estrogen receptor in the sc-PDB (2013) was assessed using binding site patches created with varying *site sizes* (Additional file 1: Figure S1). Larger binding site patches incur a penalty in calculation time during BSC, and therefore the chosen *site size* represents a balance between computational expense and retrieval success.

The SiteHopper tool was utilized to generate binding site patches and to assess pairwise structure similarity between reference and query patches. The default SiteHopper *PatchScore* represents a summation of Tanimoto similarity coefficients [37] weighted 3:1 in favor of color similarity over shape similarity, yielding a continuous value between zero and four, conveying complete dissimilarity and perfect similarity respectively [13]. Utilization of the symmetric Tanimoto similarity coefficient causes an inherent size matching to exist between pairs of binding site patches that show high levels of structural similarity.

### Retrieval analysis

The sc-PDB (2013) database [23] was utilized to assess BSC performance through a series of retrieval experiments evaluating the ability of SiteHopper to identify similar binding sites belonging to the same protein target. True positives were defined as binding site patches with the same UniProt identifier as the query patch, except for those belonging to HIV-1 protease, which were defined by sequence searching as previously described (“Methods” section). Due to the presence of multiple binding sites per protein structure, only the binding site with the highest SiteHopper PatchScore derived from a matching protein structure was considered a true positive. Reference binding site patches used as queries for retrieval experiments are shown in Additional file 1: Table S1.

### Mapping binding sites

To guide mapping of the potentially ligandable binding sites of the structural proteome, an exhaustive all-against-all BSC was performed on the Pilot dataset containing 2708 binding sites generated by fpocket. A breakdown of the Pilot dataset, including the number of binding sites detected for each protein target, is shown in Additional file 1: Table S2. The resulting matrix of (2708 × 2708) SiteHopper PatchScores was exploited to produce a clustered heat map of potential binding site space. To remove non-conserved binding sites from the dataset, patches with fewer than five pairwise SiteHopper PatchScores ≥2.0 were filtered out. Binding sites were first clustered within the protein targets from which they were derived using average-linkage agglomerative hierarchical clustering and the Euclidean distance measure. Subsequent clustering was performed in the same way across the global Pilot dataset. Plots were generated using *matplotlib* [38] and clustering was implemented in the *Python* programming language using the *SciPy* package [39].

*Density*-*Based Spatial Clustering of Applications with Noise* (DBSCAN) [40] was applied to cluster binding sites for each protein using *n* × *n* matrices of SiteHopper PatchScores. DBSCAN was implemented in the Python programming language using the *scikit*-*learn* machine learning toolkit [41] with range *ε* = 7 and a minimum number of points per core cluster being ten. Circular plots were generated as an alternative visual tool for mapping potential binding site space using the *Circos* software package [42].

### Evaluation techniques

*Receiver Operating Characteristic* (ROC) curves are a widely used tool employed to quantify the ability of a method to identify instances with similar characteristics to a reference (true positives). The *Area Under a Receiver Operating Characteristic* (AUROC) curve provides a measure of how well a method distinguishes between true positives and false positives in a dataset [43]. A perfect separation of all true positives from the data would result in an AUROC of 1, whilst a random classifier would be expected to distribute true positives throughout the whole dataset resulting in an AUROC of 0.5.

Often, it is the early recognition of true positives that is important [44], especially in cases where *n* is large and AUROC results are indistinguishable between methods. To this end, *Enrichment Factors* (EF) at 5% and the *Boltzmann*-*Enhanced Discrimination Receiver Operating Characteristic* (BEDROC) [45] were also calculated. An EF is the ratio of the percentage of true positives in an initial portion of a dataset, to the overall percentage of true positives in the entire dataset. Thus an EF = 1 implies no enrichment in the initial portion of the data (no early enrichment); EF < 1 implies the classifier performs worse than random at identifying true positives, and EF > 1 implies there is some quantifiable enrichment of true positives among the highest ranked data points [46]. BEDROC applies Boltzmann weighting to the AUROC calculation thereby emphasizing the initial portion of the ROC curve—calculated using the *CROC* package [45] at α = 20 [46, 47].

Throughout this study, the *OEChem* chemoinformatics toolkit [48] was used as an interface between tools and data was handled using the *pandas* data analysis framework [49].

## Results and discussion

### Ligand-independent binding site detection

Typically, BSC studies make use of known binding sites characterized by surface protein atoms surrounding an experimentally observed bound ligand. To objectively consider currently unliganded binding sites, ligand-independent binding site detection tools were evaluated. Incorporation of binding site detection tools removes bias associated with utilizing currently known liganded binding sites; however, it may also introduce noise to the data through inclusion of cavities that are incapable of ligand binding. Therefore, an assessment of the noise introduced to the data by binding site detection was conducted and also of the subsequent implications for BSC performance.

fpocket is a well-established and freely available binding site detection tool capable of operating in high-throughput and therefore applicable to large datasets of protein structure data [e.g. the sc-PDB (2013) contains 9283 structures]. fpocket was evaluated according to three criteria: Its ability to (1) detect cavities corresponding to functionally relevant binding sites starting from a global search of a protein structure; (2) detect similar cavities from an ensemble of structurally similar experimental structures of the same protein bound to the same ligand; and (3) rank and prioritize detected cavities according to their likelihood of binding small molecule ligands. Two datasets were utilized to assess these criteria: an ensemble of five ATP-bound cAMP-dependent protein kinases, and the PDBBind-refined set (2014) [29].

**Fig. 2:**
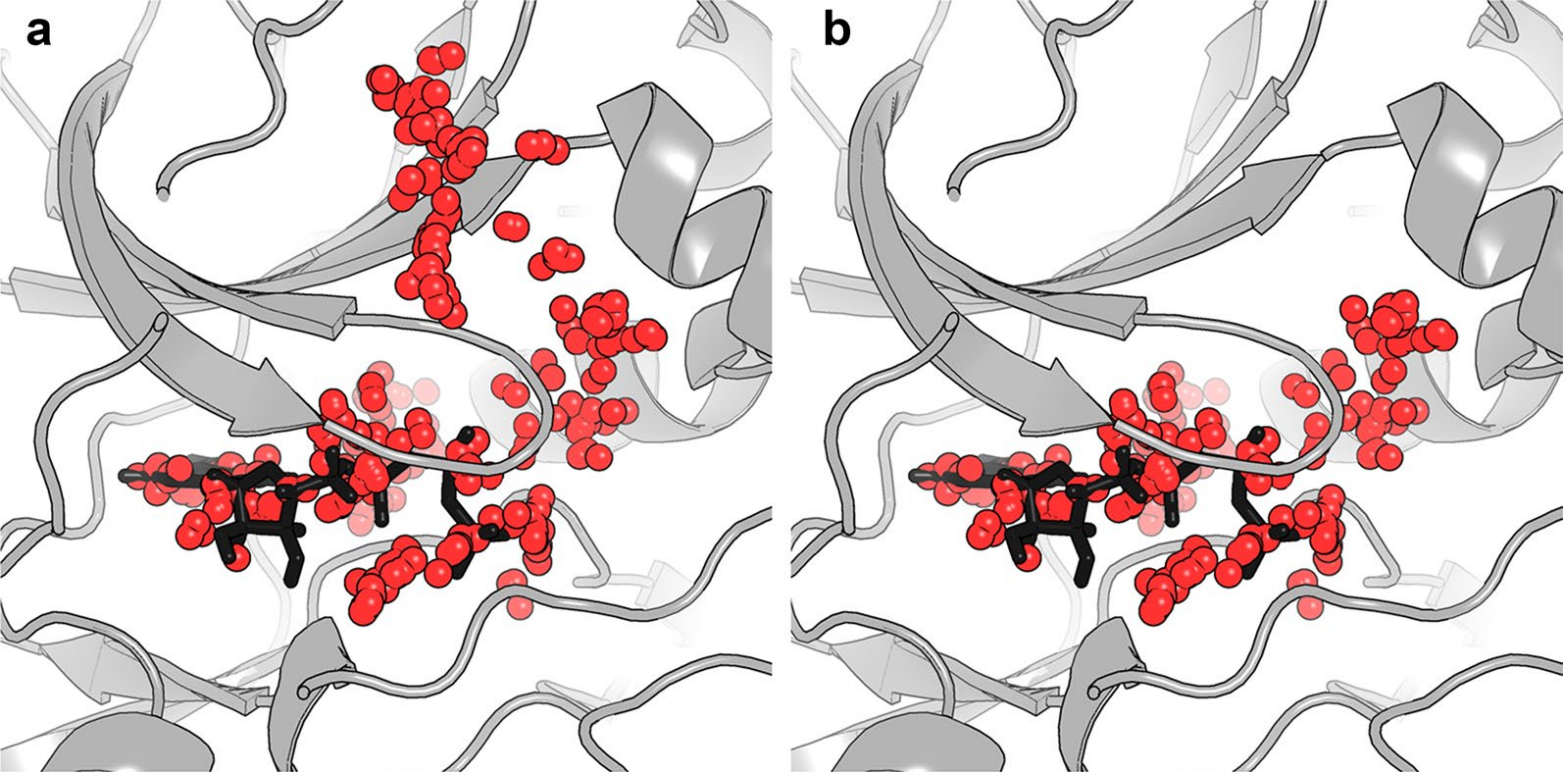
fpocket cavity detection exemplified for the ATP-binding site of a single ATP-bound cAMP-dependent protein kinase (PDB ID: 1ATP), ATP shown as *black* sticks. Alpha spheres corresponding to binding sites detected by fpocket are shown in *red* for **a** default, and **b** modified parameters

Initially, fpocket was evaluated qualitatively on a model ensemble of five structurally similar ATP-bound cAMP-dependent protein kinases for its ability to detect a cavity corresponding to the well-characterized ATP-binding site. fpocket implemented with default parameters tended to identify cavities extending beyond the ATP-binding domain. To attenuate this phenomenon, default parameters were modified to prevent the merging of distinct sub-pockets, yielding more concisely defined and consistent cavities amenable to BSC studies. The difference between default and modified fpocket parameters for an exemplar protein structure is shown in Fig. 2a, b, respectively.

**Fig. 3:**
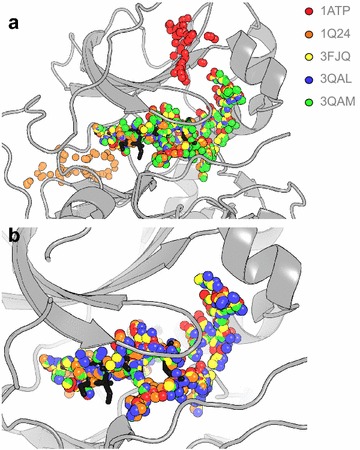
fpocket cavity detection exemplified for the ATP-binding site of an aligned ensemble of five ATP-bound cAMP-dependent protein kinases; overlaid on an exemplar structure with the ATP ligand shown in *black* sticks (PDB ID: 1ATP). Alpha spheres corresponding to cavities detected by fpocket are shown for **a** default, and **b** modified parameters

Geometric binding site detection algorithms are inherently sensitive to slight variations in protein atomic coordinates. A second qualitative validation shows that fpocket is capable of identifying similar cavities for each member of the aligned cAMP-dependent protein kinase ensemble. This assessment is depicted for fpocket with both default and modified parameters in Fig. 3a, b respectively. fpocket cavities detected for PDB IDs 1ATP and 1Q24 show variation from the core ATP-binding cavity when implemented with default parameters; however, the modified parameters provided more consistent cavity representation.

fpocket binding site detection often identifies multiple cavities per protein structure. To reduce the complexity associated with carrying forward multiple cavities per protein for BSC, the detected binding sites were ranked and subsequently filtered according to their potential to bind a small molecule ligand. Many other binding site detection tools rank cavities according to binding site volume as often the largest cavity corresponds to the observed ligand binding site [50]. The fpocket *Score* model aims to predict whether a cavity may contain a bound small molecule ligand and is distinct from a drug ability model since ligands are not necessarily drug-like [8].

**Fig. 4:**
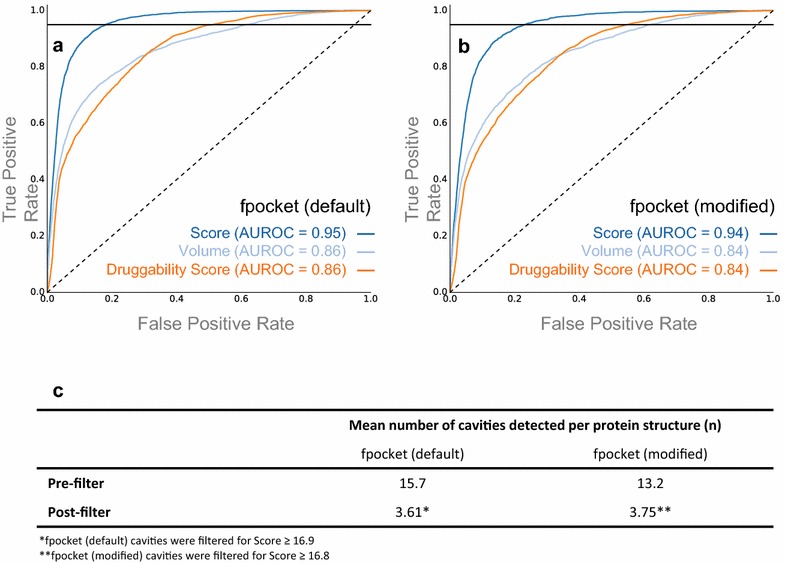
ROC curves assessing the ability of fpocket descriptors Score, Volume and Druggability Score to identify the 3446 ligand-bound binding sites derived from the PDBbind refined set (2014) over other detected cavities. *Curves* shown for fpocket **a** default parameters, and **b** modified parameters; the threshold corresponding to 95% recall is shown on each plot. **c** Mean number of cavities detected per protein structure before and after the 95% recall filter was applied for both default and modified fpocket parameters

The PDBBind-refined set (2014) [29], comprising 3446 protein structures, was processed by fpocket. The ability of the three descriptors, *Score*, *Volume* and *Druggability Score* [51], to prioritize experimentally validated ligand-binding sites over unliganded cavities was assessed. Figure 4a, b show ROC curves for fpocket with both default and modified parameters, respectively. In both cases, the PLS model *Score* was superior in discriminating between true known liganded binding sites, and those corresponding to cavities without ligands bound.

A comparison of binding site detection tools has previously found that 95% of ligand-bound sites are identifiable using geometric algorithms [7]. Accordingly, a sensitivity threshold was determined above which 95% of observed ligand binding sites from the PDBBind-refined set were identified. This sensitivity threshold precedes a sharp increase in false positive rate and therefore excluding sites below the sensitivity threshold ensures that the number of cavities without ligand-binding potential introduced to the dataset is limited. Thus, cavities were taken forward to BSC if the fpocket *Score* is ≥16.8, corresponding to the Score above which 95% of the ligand-bound cavities from the PDBBind-refined set are identified.

The average number of cavities identified *per* protein structure before and after applying the 95% recall filter is shown for both default and modified fpocket parameters (Fig. 4c). Although fpocket with modified parameters (yielding smaller, more concise cavities) performs slightly worse than default according to ROC analysis, the number of cavities detected per protein is comparable and therefore both parameter sets introduce similar levels of noise to the dataset. However, smaller and more consistent cavities are beneficial for BSC in terms of studying binding site similarity. Therefore, we elected to study BSC using cavities detected by fpocket with modified parameters; it can be assumed that further mentions of fpocket refer to this non-standard modified model.

### Binding site comparison (BSC)

Binding site patches were generated using the SiteHopper create tool, utilizing fpocket surface protein atoms as a pseudo-ligand and isolating binding site patches from the original protein structure. The BSC performance of SiteHopper was assessed for its ability to find structural similarity between a query patch and analogous patches derived from the same protein. Six protein targets of relevance to small molecule therapy were selected from the sc-PDB (2013) database as exemplar queries (Additional file 1: Table S1). To assess the impact of incorporating binding site detection into BSC, the retrieval performance of SiteHopper was first evaluated utilizing only binding site patches defined surrounding an observed bound ligand. This ligand-dependent dataset contains 9275 known binding sites and is referred to as the ligCav dataset. The sc-PDB (2013) database was also processed by fpocket to produce a dataset of 24,345 potential binding sites (including known binding sites), for which SiteHopper patches were generated. Retrieval performance was evaluated through generation of ROC curves along with two early enrichment metrics: The EF at 5% and the BEDROC at α = 20 [45]. Table 1 summarizes the ability of SiteHopper to detect structural similarity between analogous binding sites derived from structures of the same protein, for both the ligCav and fpocket-derived datasets.

**Table 1:** Evaluation of SiteHopper retrieval of binding site patches from the sc-PDB (2013) belonging to the same protein as a query patch

	N (sc-PDB)	AUROC	EF (5%)	BEDROC
ligCav				
BRD4 (*n* = 2)	15	1.00 ± 0.00	20.03 ± 0.00	1.00 ± 0.00
Carbonic anhydrase 2 (*n* = 3)	76	1.00 ± 0.00	19.77 ± 0.00	0.99 ± 0.00
CDK2 (*n* = 3)	180	1.00 ± 0.00	19.66 ± 0.07	0.97 ± 0.01
Estrogen receptor (*n* = 5)	58	1.00 ± 0.00	20.03 ± 0.00	0.96 ± 0.01
HIV-1 protease (*n* = 3)	219	0.99 ± 0.00	19.94 ± 0.00	0.99 ± 0.00
Prothrombin (*n* = 3)	126	1.00 ± 0.00	20.03 ± 0.00	1.00 ± 0.00
fpocket				
BRD4 (*n* = 2)	15	0.97 ± 0.03	18.67 ± 1.33	0.94 ± 0.06
Carbonic anhydrase 2 (*n* = 3)	76	0.99 ± 0.00	18.69 ± 0.00	0.93 ± 0.00
CDK2 (*n* = 3)	180	0.76 ± 0.03	8.83 ± 1.74	0.43 ± 0.10
Estrogen receptor (*n* = 5)	58	0.94 ± 0.01	16.07 ± 0.17	0.80 ± 0.01
HIV-1 protease (*n* = 3)	219	0.98 ± 0.00	19.52 ± 0.00	0.97 ± 0.00
Prothrombin (*n* = 3)	126	0.94 ± 0.01	17.76 ± 0.32	0.88 ± 0.01

Comparison between the ligCav dataset of ligand-dependent binding sites (*N* = 9275), and those identified through fpocket cavity detection (*N* = 24,345). Mean results and standard errors are shown. *n* = number of queries; for further detail of the query and true positive patches see Additional file 1: Table S1

As described above, the incorporation of fpocket cavity detection into BSC introduces the potential for noise in the binding site dataset compared to only defining binding sites surrounding observed bound ligands, and this may result in poorer retrieval performance metrics. However, in our retrieval analysis, we only observed a slight impact on BSC performance using early enrichment metrics; the AUROC enrichment remains high when compared to retrieval analyses performed using the ligCav dataset. Thus, the incorporation of fpocket objective cavity detection into BSC workflows is not associated with an unreasonable decrease in retrieval capability. In summary, we show that SiteHopper is able to identify structural similarity between potential binding sites that have been detected objectively from protein structure coordinates.

Interestingly, we observed a variation in retrieval rates across protein targets. Retrieval scores for the acetyl-lysine binding site of BRD4 are high, likely due to the rigidity of the protein structure surrounding this site. On the contrary, EF at 5% and BEDROC for the protein kinase CDK2 are relatively poor, likely due to the flexibility and range of protein conformations exemplified by crystal structures of this protein. Upon inspection of instances where structural similarity was expected, but not assigned a high SiteHopper score, we found that, in many cases, analogous fpocket-detected potential binding sites showed structural variability. This observation highlights the importance of consistency in the binding site detection tool; for example, upon inspection of cavities detected for prothrombin, we found that overlapping but dissimilar fpocket-detected sites were extracted from very similar protein conformations. This exemplifies how the objective implementation of fpocket binding site detection can still introduce noise into the BSC workflow despite the modifications we describe.

### Clustering and mapping of potential binding sites

We applied the Pilot dataset to develop and validate a method for clustering and mapping objectively detected potentially ligandable binding sites. An exhaustive all-against-all SiteHopper BSC was performed to generate a 2708 × 2708 matrix of SiteHopper PatchScores. Possible PatchScores range from zero to four, where zero indicates total pairwise dissimilarity and four indicates perfect similarity. To remove non-conserved, information-poor potential binding sites, those with fewer than five PatchScores ≥2.0 were removed. Starting from an initial pool of 2708 potential binding sites, this criterion reduced the data to a 1706 × 1706 matrix of SiteHopper PatchScores.

**Fig. 5:**
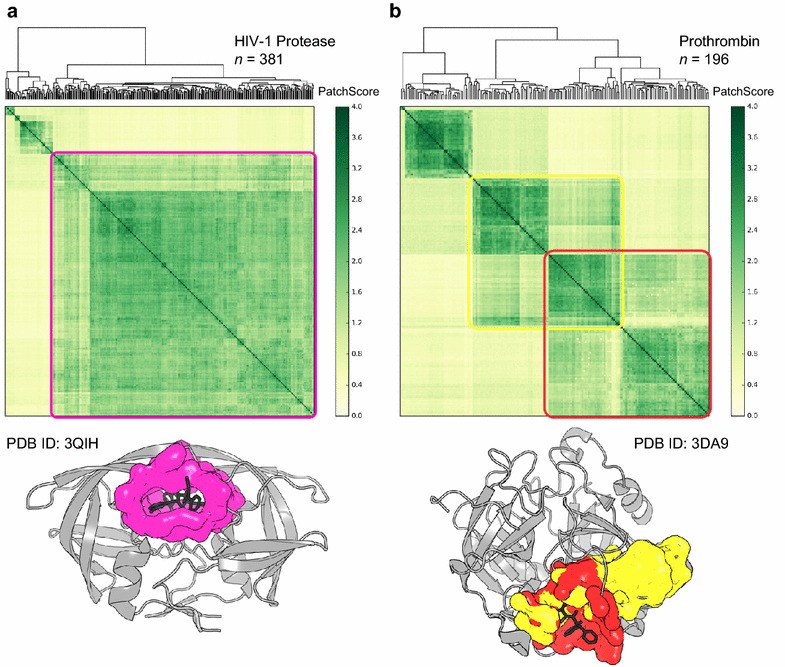
Heat map showing clustering of SiteHopper PatchScores within proteins of the same target class for potential binding sites of **a** HIV-1 Protease, and **b** prothrombin. Clustering patches derived from structures of the same protein target identifies conserved potential binding sites; the locations of highlighted exemplar clusters are shown on representative protein structures below each heat map

Hierarchical clustering provides an objective method for grouping structurally similar binding sites. Potential binding sites were first clustered locally according to their SiteHopper PatchScores across all available structures of the same protein target. Binding sites that share a pattern of similarity across the dataset are clustered together and represent groups of distinct potential binding sites within the protein family. Clusters vary in size, indicating that some cavities are more conserved than others. For all protein targets in the Pilot dataset, the largest cluster for each protein corresponds to the conserved, orthosteric ligand-binding site. Figure 5 shows exemplar clustered heat maps of SiteHopper PatchScores for HIV-1 protease and prothrombin with the location of highlighted exemplar clusters shown on representative protein structures below each heat map. The most conserved cluster of potential binding sites for HIV-1 protease corresponds to the protease catalytic binding site to which many compounds are known to bind. Interestingly, binding site similarity is identified despite the prevalence of binding site mutations among structures of this protein. The large volume of the HIV-1 protease binding site, such that each pharmacophoric pseudocenter contributes less to the overall similarity, may contribute to the homogeneity of this conserved cluster. Figure 5b highlights two distinct prothrombin potential binding sites that show a degree of structural similarity to each other. This is a phenomenon arising from the integration of cavity detection into the workflow and represents a case where two overlapping binding sites are detected (depicted in red and yellow).

**Fig. 6:**
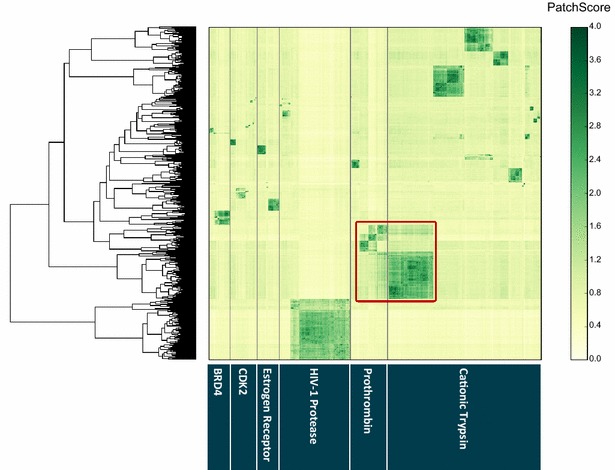
Heat map of SiteHopper PatchScores across the Pilot dataset of 1085 protein structures represented by 1706 conserved potential binding sites. Binding sites are grouped within individual protein targets on the x-axis and a global hierarchical clustering is shown on the y-axis. A similarity score of 0 and 4 indicates complete dissimilarity and perfect similarity, respectively. Similarity between potential binding sites derived from different protein targets, cationic trypsin and prothrombin, is highlighted in *red*

Next, we applied hierarchical clustering globally to the Pilot dataset, grouping clusters of similar binding sites regardless of their parent protein target. A combination of clustering both locally within protein targets and globally across the entire Pilot dataset generates a map of potential binding site space (Fig. 6).

The number of dominant clusters in the Pilot dataset represents the homogeneity of potential binding sites across the six proteins (Fig. 6). The scarcity of clusters of conserved binding sites among CDK2 structures is consistent with the flexibility of this kinase observed in protein crystal structures—notably the presence of diverse active and inactive protein conformations. Notably, SiteHopper identifies dominant clusters of substantially conserved binding sites for each of the five other protein targets. The most highly conserved binding site is that of the HIV-1 protease, likely due to the large volume and enclosed shape of the catalytic binding site that enables consistent identification by fpocket and robust detection of similarity by SiteHopper, respectively. Other factors that will likely affect the presence or absence of conserved binding sites within available structures of a particular protein include the presence of apo and holo bound structures, particularly for proteins containing multiple domains [52].

The global cluster analysis highlights a region of overlap between two clusters of binding sites belonging to prothrombin and cationic trypsin (Fig. 6). SiteHopper identifies structural similarity between the catalytic protease binding sites of these proteins (highlighted in red, Fig. 6). These two proteins are known to bind similar compounds and are annotated with a *selectivity group* of 254 compounds in the ChEMBL database (version 21) [53, 54] of bioactive molecules. Compounds in the selectivity group represent literature examples where the ratio of binding (selectivity coefficient) between prothrombin and trypsin has been measured; 99 of the 254 selectivity group examples exhibit a selectivity ratio of less than ten indicating that ligands commonly bind to both protein targets. The identification of similarity between these binding sites exemplifies the potential of BSC tools to rationalize and predict polypharmacology independent of ligand data.

Despite efforts to minimize the noise introduced by cavity detection, non-conserved potential binding sites inevitably affect the interpretability of clustered heat maps because much of the heat map conveys regions of structural dissimilarity—which is less informative than similarity. Furthermore, non-conserved binding site patches that do not show SiteHopper similarity to other patches are grouped together by clustering methods, generating a group of information-poor binding sites. One method to reduce the presence of these information-poor binding sites is to apply stricter binding site conservation criteria. However, these would penalize potentially interesting novel proteins for which there are fewer instances exemplified in the PDB versus more extensively studied proteins.

**Fig. 7:**
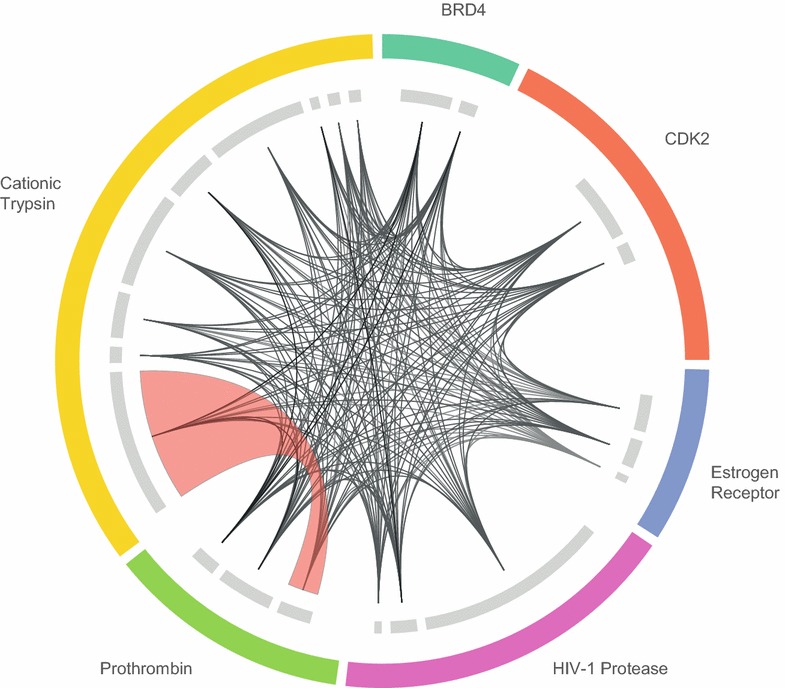
Circular map of potential binding site space within the Pilot dataset. Protein targets are ordered alphabetically on the *outer circle*, and binding site clusters identified by DBSCAN for each protein are indicated by *gray bands*. Links between groups of distinct protein binding sites represent the maximum PatchScore observed between members of those groups. Links associated with a PatchScore ≥2.0 are shown as a *red ribbon*. Plot generated by Circos [42]

DBSCAN [40] is a clustering algorithm widely used in data science that aims to group closely related points, and to label those points with few neighbors as noise. DBSCAN is suited to binding site analysis because it is not designed to create uniformly sized clusters [55]. DBSCAN was implemented to cluster potential binding sites locally within protein targets for the Pilot dataset. Due to the high dimensionality of the underlying data, visualization of DBSCAN clustering has proven challenging (the variance explained by the first two principal components is 0.42 and 0.18, respectively). Here we present a circular plot to map DBSCAN clustering of potential binding sites in the Pilot dataset, with protein targets arranged on the outer circle (Fig. 7). Conserved binding site clusters associated with each protein target are represented by gray bands on the inner circle; links describing the structural similarity between two binding sites are shown in the center. Each link is associated with the maximum SiteHopper PatchScore exhibited by members of two binding site clusters and those associated with a SiteHopper PatchScore ≥2.0 are highlighted as red ribbons. Consistent with the global cluster analysis (Fig. 6), this approach also highlights a structural similarity between binding sites belonging to prothrombin and cationic trypsin confirming the ability of BSC tools to identify the potential for ligand polypharmacology.

## Conclusions

In constructing a workflow to map the binding sites of the currently characterized structural proteome, we adopted a modular approach that comprises objective binding site detection, binding site comparison (BSC), mapping of detected binding sites using unsupervised learning methods, and visualization of binding site maps. Although we outline a workflow for mapping potential small molecule binding sites in proteins, each of the components can be altered according to the tools available and specific hypothesis under test.

We applied fpocket as a geometric cavity detection tool to identify potentially novel unliganded binding sites, and modified fpocket parameters to yield concise cavities that are better suited to subsequent BSC studies. To filter out fpocket cavities that are unlikely to be ligandable, we determined a threshold fpocket *Score* by analyzing retrieval rates from the PDBbind-refined set; cavities were taken forward to BSC if the fpocket Score is ≥16.8, corresponding to the Score above which 95% of the ligand-bound cavities from the PDBBind-refined set are identified.

Applying fpocket cavity detection to the sc-PDB dataset (2013) to assess the impact of incorporating objective and unbiased cavity detection to BSC compared with only defining binding sites that surround exemplified bound ligands. Using SiteHopper for BSC, we show that the penalty associated with replacing ligand-dependent binding sites with objectively detected cavities is minimal and importantly also allows consideration of currently unliganded sites in BSC studies.

The workflow we describe applies the fpocket geometric detection algorithm to detect cavities in a protein structure. A limitation is that local chemical interaction hotspots and flat binding sites that are particularly relevant for the study of Protein–Protein Interactions (PPIs), will not be identified. To map such binding sites, it may be possible to introduce an interaction hotspot prediction tool such as FTMap [56], GRID [57] or SuperStar [58] into the modular workflow; this will be the subject of future studies.

The Pilot dataset was processed by fpocket and an all-against-all SiteHopper BSC was performed to create a matrix of binding site similarities. Hierarchical clustering within protein structures derived from the same protein target reveals a large proportion of cavities that are not conserved across multiple structures of the same protein; we therefore introduced a conservation filter (removal of cavities with fewer than five PatchScores ≥2.0) to minimize the number of information-poor cavities in the dataset. A combination of clustering both locally within protein targets and globally across the entire dataset, generates a map of potential binding site space. Furthermore, we show that density-based clustering by DBSCAN is an appropriate method for generating clusters of binding sites and mitigating the noise introduced to the dataset by objective fpocket cavity detection.

Although a powerful visualization, heat maps can be challenging to interpret, and therefore we introduce circular plots as an intuitive tool for visualizing and mapping structural binding site space. We show that such plots can highlight the similarity between binding sites derived from different proteins. Here, we exemplify an objectively identified similarity between binding sites of the serine proteases prothrombin and cationic trypsin that is consistent with literature reports that their catalytic sites bind similar ligands. We suggest that such protein binding site maps will be useful for building further understanding of the relationship between small molecules and complex biological systems; this approach is potentially applicable to the discovery of hit matter for novel biological targets, for predicting and rationalizing ligand polypharmacology and for predicting protein function [3, 4]. In addition, we suggest that such an objective binding site map, which encompasses unliganded cavities, will also be useful for optimizing compound screening collections towards a more complete chemical coverage of binding site space. We will present examples of such applications in due course.

## Additional file

**Additional file 1.** A list of PDB IDs for two curated datasets utilised in this study: ATP-bound cAMP-dependent Kinase ensemble (n = 5) and the Pilot Dataset (n = 1085). **Figure S1**. Mean ROC curves for a series of experiments to determine the optimum site size to generate binding site patches surrounding fpocket surface atoms. **Table S1**. Summary of proteins from the sc-PDB (2013) that were considered in ROC retrieval studies. **Table S2**. Summary of the Pilot Dataset.

## Abbreviations

AUROC: area under receiver operating characteristic
BSC: binding site comparison
BEDROC: Boltzmann-enhanced discrimination receiver operating characteristic
DBSCAN: density-based spatial clustering of applications with noise
EF: enrichment factor
PDB: protein data bank
BRD4: bromodomain-containing protein 4
CDK2: cyclin-dependent kinase 2
ER: estrogen receptor
HIV-1: human immunodeficiency virus-1
