# Supplementary material for: Mapping the 3D structures of small molecule binding sites
Source: J Cheminform. 2016 Dec 6;8:70. doi: 10.1186/s13321-016-0180-0 (PMC5395517; doi:10.1186/s13321-016-0180-0)
Supplement: Supplementary file 1 — Additional file 1. A list of PDB IDs for two curated datasets utilised in this study: ATP-bound cAMP-dependent Kinase ensemble (n = 5) and the Pilot Dataset (n = 1085). Figure S1. Mean ROC curves for a series of experiments to determine the optimum site size to generate binding site patches surrounding fpocket surface atoms. Table S1. Summary of proteins from the sc-PDB (2013) that were considered in ROC retrieval studies. Table S2. Summary of the Pilot Dataset. [file 13321_2016_180_MOESM1_ESM.pdf]

Mapping the 3D Structures of Small Molecule Binding Sites – Supporting Information

Joshua Meyers, Nathan Brown and Julian Blagg**

Cancer Research UK Cancer Therapeutics Unit, Division of Cancer Therapeutics, The Institute of Cancer Research, London, SM2 5NG, UK.

Supporting Information

Protein Structure Datasets

Dataset	PDB IDs
ATP-bound cAMP-dependent Kinase ensemble	1ATP, 1Q24, 3FJQ, 3QAM, 3QAL
Pilot Dataset	BRD4: 4F3I, 4NQM, 4E96, 3UVW, 3UVX, 3UVY, 4GPJ, 4LZS, 4C67, 5BT4, 4XY9, 4QZS, 3P5O, 4HBX, 4HBY, 4HBV, 4HBW, 4XYA, 4IOQ, 4IOR, 4IOO, 4J3I, 3SVG, 4A9L, 3UW9, 4UIZ, 4QB3, 2OUO, 2YEM, 4NUE, 4UIX, 4UIY, 4PS5, 4NUC, 2OSS, 4J0R, 4J0S, 4OGJ, 4OGI, 4UYD, 4MEN, 4MEO, 4MEQ, 4WIV, 4BJX, 4MR4, 4MR3, 3U5L, 3U5K, 3U5J, 4Z1S, 3ZYU, 4KV4, 4KV1, 4CFL, 4O74, 2YEL, 4BW2, 4BW1, 4BW4, 4MEP, 4HXK, 4HXL, 4HXM, 4HXN, 4HXO, 4HXP, 4HXR, 4HXS, 5A85, 3SVF, 4LYW, 4NUD, 3MXF, 4LR6, 4LYI, 4CL9, 4QR4, 4O78, 4O76, 4O77, 4O72, 4O70, 4O71, 4PCE, 4CLB, 4Z93, 4PCI, 4O7F, 4O7E, 4O7B, 4O7C, 4O7A<br><br>CDK2: 2C69, 2C68, 4BZD, 2C6M, 2C6K, 4ACM, 3TIY, 2WEV, 4EK4, 4EK5, 4EK3, 4I3Z, 4EK8, 3R83, 4FKJ, 4FKI, 4FKO, 4FKL, 4FKG, 1V1K, 4FKS, 4FKR, 4FKQ, 4FKP, 4FKW, 4FKV, 4FKU, 4FKT, 2V0D, 3R8V, 3MY5, 3R8P, 4D1Z, 4D1X, 4EOQ, 4EOR, 4BCO, 4BCN, 4BCK, 2BHE, 4BCP, 1GZ8, 2C5N, 2C5O, 3R7Y, 1H27, 2UZL, 3NS9, 2VTP, 2VTQ, 2VTR, 2VTS, 2VTT, 2VTH, 2VTI, 2VTJ, 2VTL, 2VTN, 2VTO, 2VTA, 2A4L, 3TNW, 1H1P, 1Y91, 5A14, 1HCL, 3QTS, 4BGH, 2R64, 2UZE, 3EZR, 3LFN, 3DDQ, 1W8C, 3LFS, 3LFQ, 1OIU, 1HCK, 4II5, 4BCQ, 2VV9, 3SW7, 3SW4, 2CJM, 3BHV, 3BHU, 3BHT, 1KE7, 1KE6, 1KE5, 1KE8, 1Y8Y, 4CFU, 2VU3, 1H07, 1H01, 1H00, 1H08, 4RJ3, 1H0V, 4EK6, 5D1J, 4KD1, 3QTU, 1W98, 2BTS, 3PJ8, 3LE6, 4CFV, 3RPR, 3RPY, 4CFN, 1W0X, 2R3R, 2R3I, 2R3Q, 2R3K, 2R3J, 2R3M, 2R3L, 2R3O, 2R3P, 2R3G, 3R9N, 2W05, 2W06, 1URW, 2R3H, 2XMY, 3QQL, 1AQ1, 3QHW, 2R3N, 3UNK, 2R3F, 3IG7, 3IGG, 1PXJ, 4NJ3, 3RAL, 3RAH, 4GCJ, 1B38, 1B39, 2UUE, 3WBL, 1JVP, 2CLX<br><br>Estrogen Receptor: 4IW8, 4IW6, 4MGA, 4MGC, 4MGD, 4IWF, 4IWC, 4MG8, 4MG9, 4MG5, 4MG6, 4MG7, 2QA8, 4JC3, 2QAB, 5AK2, 3CBM, 4Q13, 4MGB, 2QSE, 1SJ0, 2B1V, 4IVY, 4IVW, 4IV2, 2FAI, 2R6Y, 4TUZ, 2B1Z, 2OUZ, 2QR9, 2YJA, 2QGT, 2P15, 4IU7, 4TV1, 2B23, 1ZKY, 4PP6, 4PPS, 1QKT, 3Q95, 2BJ4, 3CBO, 3UU7, 3UUD, 3UUC, 3UUA, 2JF9, 4JDD, 2POG, 1L2I<br><br>HIV-1 Protease: 3BXS, 2AOJ, 2AOH, 2AOI, 4Q1X, 2IDW, 2HC0, 3I8W, 4DFG, 3ZPU, 1DAZ, 4DQF, 3K4V, 4Q5M, 4K4R, 2F3K, 4K4P, 3QIH, 3O9G, 1YTH, 3B80, 3M9F, 2QAK, 2FLE, 2QNQ, 2QNP, 2PSU, 3H5B, 2QNN, 4GB2, 3EM6, 1K2B, 3LZV, 3KDB, 3KDC, 3KDD, 4Q1W, 2QI3, 4DQH, 3QRS, 4DQE, 4DQC, 4DQB, 3QRO, 3QRM, 3S54, 3S56, 3S53, 1A30, 3B7V, 3EKX, 3JVY, 1ZBG, 3EKP, 3EKW, 1EBW, 4A4Q, 2Q3K, 1EBZ, 2QD8, 3SA3, 2PWC, 3EKQ, 2PWR, 3FX5, 4LL3, 1ZP8, 3DK1, 2NMZ, 2NMY, 3EL9, 3CYW, 3EL4, 3CYX, 3EL0, 2BQV, 3OY4, 3I7E, 2QMP, 1EBY, 4HLA, 4U8W, 1B6K, 1B6J, 1B6M, 1B6L, 1B6P, 3NDU, 4FL8, 3ZPT, 3ZPS, 1U8G, 3R4B, 2QI0, 2QI1, 3EM4, 2QI4, 2QI5, 2QI6, 2QI7, 3EM3, 3QN8, 3DJK, 3D1Z, 5AHB, 5AHC, 5AHA, 1Z8C, 2FXE, 3D1Y, 3KT5, 4KB9, 3NU3, 1ZSR, 3BVA, 3BVB, 4TVG, 1ZSF, 2Z4O, 2Q63, 4EJ8, 5AH6, 5AH7, 3JV

W, 5AH8, 5AH9, 3NLS, 4EJD, 4EJL, 4EJK, 3QAA, 2PYM, 3EL5, 2B60, 3VFA, 3VFB, 4CPR, 1G2K, 4ZLS, 2HS1, 3VF5, 1XL2, 3VF7, 1ZTZ, 1XL5, 3CKT, 2I0A, 3BC4, 2I0D, 3GI5, 3GI4, 3GI6, 2F80, 2F81, 2G69, 5AGZ, 2Q5K, 4HEG, 1EC3, 1EC2, 1EC1, 1EC0, 4HDF, 4HDB, 2F8G, 4HDP, 3BHE, 1SDT, 1SDU, 1SDV, 3NU4, 3NU5, 3NU6, 4TVH, 2PC0, 2AOF, 1LZQ, 4ZIP, 3KFR, 3KFS, 3PSU, 3QP0, 4FLG, 3KFN, 2R38, 2AVV, 2AVQ, 2AVS, 2AVM, 2AVO, 2WL0, 3NUO, 3NUJ, 1IIQ, 3KF0, 2NNP, 3QPJ, 4FM6, 3TLH, 3KT2, 2FGU, 1D4L, 3BGB, 3BGC, 3QBF, 2QCI, 3D20, 4I8W, 4I8Z, 2WHH, 1D4J, 2WKZ, 2QHZ, 2QHY, 4DJO, 4DJR, 4DJP, 4DJQ, 2PQZ, 1YTG, 3PWM, 3PWR, 2Z54, 2PSV, 1NH0, 4EPJ, 1WBM, 1WBK, 3TKW, 4OBF, 1LV1, 4OBH, 4OBJ, 4QJA, 3OK9, 4EP3, 4EP2, 2NNK, 2HB4, 1G35, 2HB2, 2HB3, 1AJV, 1AJX, 4U7V, 3SA9, 3SA8, 4E43, 3SA5, 3SA4, 3SA6, 4HE9, 1D4K, 2A1E, 1D4I, 1D4H, 2IEN, 2IEO, 3OXC, 2R3T, 2R3W, 3SAA, 3SAC, 3SAB, 3OXV, 3OXW, 2R43, 2AQU, 4CP7, 3OXX, 3ST5, 2XYE, 2XYF, 4CPS, 4CPQ, 4FE6, 4CPW, 4CPT, 3LZS, 4A6C, 4A6B, 3TL9, 3JW2, 1SP5, 2CEN, 2CEM, 3T11, 3TOH, 3TOF, 3TOG, 3D1X, 3DOX, 1W5Y, 1W5X, 1W5W, 1W5V, 2PYN, 3MXD, 3MXE, 4MC6, 4MC1, 4MC2, 1Z1R, 3S43, 4MC9, 3TH9, 1Z1H, 4F76, 4F75, 4F74, 4F73, 1ZJ7, 1DW6, 2HS2, 2QD7, 3I6O, 3TKG, 2P3B, 2P3A, 3O99, 3BXR, 1KZK, 2QD6, 2AZC, 2ZGA, 2AZ8, 3O9E, 3O9D, 4Q1Y, 3O9F, 3O9A, 3O9C, 3O9B, 3O9I, 3O9H

Thrombin: 3LDX, 3F68, 3BIV, 3QX5, 2A0Q, 4CH8, 4CH2, 3U9A, 4RKO, 3D49, 3BEI, 2GP9, 1SFQ, 3U69, 2B5T, 2OD3, 2C93, 4LZ1, 3TU7, 2JH0, 2ZHQ, 2JH5, 2JH6, 3SQE, 3U8O, 3SHC, 3SHA, 3GIS, 2ZHE, 2ZHF, 1XMN, 4MLF, 2ZHW, 4N3L, 3SV2, 2PW8, 3VXE, 3BIU, 3NXP, 2ZFF, 2ZFQ, 4DII, 4DIH, 2ZF0, 2ZC9, 3DA9, 4LOY, 3UWJ, 3RM0, 2CN0, 3RM2, 1BCU, 3RMM, 3RMO, 3RMN, 3S7K, 1BHX, 1O5G, 2ZIQ, 2R2M, 1EOJ, 2C8W, 2C8Y, 2C8X, 2C8Z, 3DD2, 3S7H, 3QWC, 3P17, 3UTU, 3SI3, 2ZDV, 3BV9, 2ZNK, 3JZ1, 4LXB, 3DHK, 3U98, 2ZO3, 2H9T, 3C27, 1TQ7, 2CF9, 2CF8, 2ZG0, 3RLW, 3RLY, 4YES, 4H6S, 2ZGX, 1SHH, 4AX9, 3RML, 1VZQ, 3QTO, 3QTV, 2PGB, 2AFQ, 1DOJ, 1G30, 3DUX, 1G32, 2ZDA, 1YPL, 1YPM, 1YPJ, 1YPK, 1YPG, 1YPE, 1GJ5, 4BAM, 3SI4, 3T5F, 3U8R, 3U8T, 2UUJ, 3EGK, 4BAN, 1KTT, 4BAK, 3P6Z, 4BAH, 4E7R, 1Z8I, 1Z8J, 1DE7, 3EQ0, 3R3G, 1EOL, 4NZE, 2ZFR, 1O2G, 1H8I, 2V3H, 2ZI2, 1SB1, 3QLP, 4RKJ, 3K65, 1OYT, 2ZGB, 4DT7

Trypsin: 3RXO, 3A84, 3RXM, 3RXL, 3RXJ, 3RXG, 3RXF, 3RXD, 3RXC, 3RXA, 3BTF, 1V2T, 3NK8, 4NIX, 3RXK, 3RXU, 1O3K, 1Y59, 3RXQ, 3RXP, 1XUI, 1RXP, 3GY3, 1Y5A, 3NKK, 1Y5B, 4Y10, 2J9N, 3A89, 1Y5U, 1O3M, 1O35, 1O37, 4Y11, 1O36, 1F2S, 1O3L, 3V0X, 3ATK, 3ATL, 3AAU, 3MFJ, 3V12, 3RXB, 3RXV, 3UQO, 3RXT, 1HJ9, 3RXS, 1O3N, 3UNS, 3UOP, 3Q00, 3UNQ, 1UTO, 2BLV, 2BLW, 3GY8, 3A85, 4ABB, 1GI4, 1V2W, 3D65, 1CE5, 1O3C, 1O3B, 1O3E, 1O3F, 3RU4, 3GY4, 2ZFS, 1O30, 1O33, 1O32, 1O39, 1O2O, 1JIR, 1UTN, 3GY2, 3ITI, 2BZA, 3QK1, 3A82, 1GHZ, 2G8T, 3A83, 4KTU, 4KTS, 3RXI, 3BTH, 3T26, 2FI4, 2FI3, 1QBO, 2G81, 4XOJ, 2FTL, 2FTM, 3T27, 3A8C, 1G3D, 3MI4, 1C5P, 1J8A, 1V2J, 3BTD, 3RXE, 3A88, 2PLX, 1C2M, 4AOQ, 4AOR, 1OPH, 2D8W, 3V13, 1D6R, 3RXH, 3ATM, 3PYH, 4AB9, 4AB8, 2A7H, 3UPE, 4HGC, 2AYW, 1C1N, 1UTQ, 3RDZ, 4ABI, 1C1S, 1C1R, 1C1Q, 1C1P, 2ZHD, 4ABA, 4ABG, 4ABF, 4ABE, 4ABD, 1C5Q, 3PLP, 1TIO, 2O9Q, 1QCP, 4B2C, 2AH4, 1O31, 2ZDK, 4ABJ, 1XUG, 3A87, 3A80, 3A81, 3VPK, 1GI3, 1GI1, 1GI0, 1GI6, 1GI5, 4NIV, 1V2S, 1O2W, 1O2T, 1V2Q, 3A8D, 1V2P, 3A8A, 3A8B, 1G36, 1C5S, 1C5R, 1C1T, 1C5V, 1C5U, 1C5T, 3GY5, 3ATI, 3PLB, 3UQV, 2BY7, 1NC6, 2TIO, 3PM3, 4Y0Y, 3UWI, 1V2K, 4Y0Z, 1Y3W, 1Y3V, 1Y3U, 1Y3Y, 1O2M, 1O2J, 2AGE, 3PMJ, 1V2N, 3A86, 4YTA, 1V2M, 2ZQ2, 1C2G, 1C2K, 1C2H, 1C2I, 1C2L, 1Y3X, 2ZDL, 1O34, 2CMY, 1V2O, 2ZDM, 2AGG, 1OX1, 4I8J, 3AAS, 4U2W, 1P2I, 1P2J, 3PWB, 3PWC, 2G55, 2OTV, 3LJJ, 1O38, 3T29, 1G3C, 1G3B, 1G3E, 2BY6, 1GJ6, 2ZQ1, 3LJO, 3GY7, 2UUY, 2G5V, 3AAV, 2ZDN, 4NIW, 1YP9, 2G5N, 4ABH, 2BTC, 1EJM, 2AGI, 1C2J, 3M7Q, 1GI2, 1O3J, 3A7Y, 2FX6, 2ILN, 2FI5, 3A7X, 1XUK, 3A7V, 3GY6, 3A7T, 3A7W, 2FX4, 1TX8, 3BTM, 3PLK, 3BTG, 3BTQ, 3BTW, 2XTT, 3BTT, 1AQ7, 3UNR, 3M35, 3A7Z, 3UUZ, 4B2A, 1V2L, 1V2U, 1XUJ, 3T25, 1C1O, 1O2S, 4TPY, 4GUX, 1O2P, 4B1T, 1G9I, 1O3G, 1O3I, 1O3H, 2ZFT, 1O2V, 1O2U, 1O2R, 1O2Q, 1O2Z, 1O2X, 1O2Y, 4MTB, 3RXR, 1O3O, 1O2N, 1O2K, 1O2H, 1O2I, 1O3D, 4J2Y, 4B2B, 2BY9, 2BY8, 2BY5, 1XUH, 4NCY, 3BTK, 1V2V, 2BYA, 1OYQ, 1SBW

---

*The overlap between the Pilot dataset and others utilized in this study are as follows: sc-PDB (2013) (n = 9275) and the Pilot Dataset (n = 1085) have 327 structures in common, PDBbind (n = 3446) and the Pilot Dataset (n = 1085) have 301 structures in common.

Figure S1. Mean ROC curves for a series of experiments to determine the optimum site size to generate binding site patches surrounding fpocket surface atoms. Five retrieval analyses identifying Estrogen Receptor binding sites from the sc-PDB (2013) were performed constituting 0.1 Å increments between 0.1-0.6 Å; 0.3 Å represents an optimal balance between computational expense and performance.

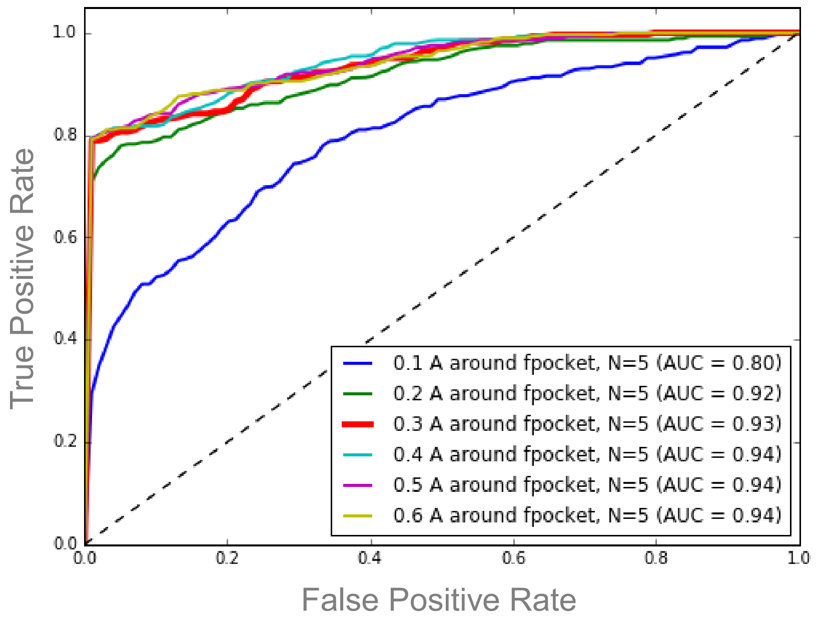

Table S1. Summary of proteins from the sc-PDB (2013) that were considered in ROC retrieval studies including those that were used to generate query binding sites.

	**UniProt ID**	***N sc-PDB***	***n* query patches**	**Query PDB IDs****
BRD4	O60885	15	2	3U5L, 4HXR
Carbonic Anhydrase 2	P00918	76	3	1I8Z, 3M98, 4BCW
CDK2	P24941	180	3	1KE9, 2BTR, 4BCO
Estrogen Receptor	P03372	58	5	1L2I, 2AYR, 2IOG, 2QGW, 4IW6
HIV-1 Protease	N/A*	219	3	1HVL, 2O4P, 3QIH
Prothrombin	P00734	126	3	1JWT, 2ZFP, 1BHX

*HIV-1 Protease entries in the sc-PDB (2013) dataset were defined by a 90% sequence similarity search (Protein BLAST, E=10^{-20}) using the consensus B protease sequence retrieved from HIVdb.[32]

**Query PDB IDs were chosen as protein structures bound to ligands with a range of heavy atom counts in order to sample multiple induced fit conformations for each protein target

Table S2. Summary of the Pilot Dataset including a breakdown of the average number of cavities detected for each protein target.

	N	**UniProt ID**	***n* fpocket predicted binding sites (per PDB)**
BRD4	93	O60885	192 (2.1)
Cationic Trypsin (bovine)	315	P00918	968 (3.1)
CDK2	148	P24941	490 (3.3)
Estrogen Receptor	52	P03372	241 (4.6)
HIV-1 Protease	335	N/A*	481 (1.4)
Prothrombin	142	P00734	336 (2.4)

*HIV-1 Protease entries in the Pilot dataset were defined by a 90% sequence similarity search (Protein BLAST, E=10^{-20}) using the consensus B protease sequence retrieved from HIVdb.[32]
